# Is treatment resistant depression a different subtype of depression?

**DOI:** 10.1192/j.eurpsy.2021.137

**Published:** 2021-08-13

**Authors:** A. Fiorillo

**Affiliations:** Department Of Psychiatry, University of Campania “L. Vanvitelli”, Naples, Italy

**Keywords:** Depression, Therapy, personalization, Suicide

## Abstract

Major depression is a serious, disabling, often chronic or recurrent mental disorder affecting over 350 million people worldwide. Treatment of major depression is now conceptualized as proceeding through three phases: the acute phase, the continuation phase, and the remission phase. Patients not achieving remission after several treatment trials are defined treatment-resistant, but a debate is ongoing regarding how many trials must fail before a patient can be defined as “treatment-resistant”. It must be acknowledged that depression is a heterogeneous disease, and several personal, socio-cultural and clinical factors should be taken into account in order to develop a personalized management plan for patients with major depression. A new concept of “difficult to treat depression” has been recently proposed. According to this concept, when a complete control of the disorder is not feasible, the treatment should aim at minimizing the impact of symptoms and the side effects of treatments on patients’ daily lives. Moreover, the concept of difficult to treat depression includes the presence of co-occurring problems/ behaviours/ disorders/ situations, which can worsen the course or management of depression. The management of patients with treatment resistant depression includes the optimization of disease management, in terms of symptom control, improvement of daily functioning and of quality of life. However, an approach aiming to personalize treatment of patients with major depressive disorder and focused on the specific clinical features of each patient can be valuable for optimizing the treatment of patients with resistant depression.

**Disclosure:**

No significant relationships.

